# Cryptic *Eimeria* genotypes are common across the southern but not northern hemisphere^☆^

**DOI:** 10.1016/j.ijpara.2016.05.006

**Published:** 2016-08

**Authors:** Emily L. Clark, Sarah E. Macdonald, V. Thenmozhi, Krishnendu Kundu, Rajat Garg, Saroj Kumar, Simeon Ayoade, Kimberly M. Fornace, Isa Danladi Jatau, Abdalgader Moftah, Matthew J. Nolan, N.R. Sudhakar, A.O. Adebambo, I.A. Lawal, Ramón Álvarez Zapata, Joseph A. Awuni, H. David Chapman, Esron Karimuribo, Claire M. Mugasa, Boniface Namangala, Jonathan Rushton, Xun Suo, Kumarasamy Thangaraj, Arni S.R. Srinivasa Rao, Anup K. Tewari, Partha S. Banerjee, G. Dhinakar Raj, M. Raman, Fiona M. Tomley, Damer P. Blake

**Affiliations:** a Department of Pathology and Pathogen Biology, Royal Veterinary College, North Mymms, Hertfordshire, UK; b Department of Veterinary Parasitology, Madras Veterinary College, Tamil Nadu Veterinary and Animal Sciences University, Chennai, India; c Division of Parasitology, Indian Veterinary Research Institute, Izatnagar, Uttar Pradesh, India; d Department of Animal Breeding and Genetics, Federal University of Agriculture, Abeokuta, Ogun State, Nigeria; e Department of Parasitology and Entomology, Faculty of Veterinary Medicine, Ahmadu Bello University, Zaria, Nigeria; f School of Agriculture, Food and Rural Development, Newcastle University, Newcastle upon Tyne, UK; g Universidad Central de Venezuela, Facultad de Agronomía Instituto de Producción Animal, Av. Universidad via El Limón, Maracay, Venezuela; h Accra Veterinary Laboratory, Accra, Ghana; i Department of Poultry Science, University of Arkansas, Fayetteville, AR, USA; j Southern African Centre for Infectious Disease Surveillance, Morogoro, Tanzania; k Department of Biotechnical and Diagnostic sciences College of Veterinary Medicine, Animal resources and Biosecurity, Makerere University, Kampala, Uganda; l Department of Paraclinical Studies, University of Zambia, Faculty of Veterinary Medicine, Lusaka, Zambia; m Production and Population Health, Royal Veterinary College, North Mymms, Hertfordshire, UK; n National Animal Protozoa Laboratory & College of Veterinary Medicine, China Agricultural University, Beijing, China; o CSIR-Centre for Cellular and Molecular Biology, Hyderabad, India; p Augusta University, Augusta, GA, USA; q Department of Animal Biotechnology, Madras Veterinary College, Tamil Nadu Veterinary and Animal Sciences University, Chennai, India

**Keywords:** *Eimeria*, Chicken, Genetic diversity, Operational taxonomic units, Vaccine

## Abstract

The phylum Apicomplexa includes parasites of medical, zoonotic and veterinary significance. Understanding the global distribution and genetic diversity of these protozoa is of fundamental importance for efficient, robust and long-lasting methods of control. *Eimeria* spp. cause intestinal coccidiosis in all major livestock animals and are the most important parasites of domestic chickens in terms of both economic impact and animal welfare. Despite having significant negative impacts on the efficiency of food production, many fundamental questions relating to the global distribution and genetic variation of *Eimeria* spp. remain largely unanswered. Here, we provide the broadest map yet of *Eimeria* occurrence for domestic chickens, confirming that all the known species (*Eimeria acervulina*, *Eimeria brunetti*, *Eimeria maxima*, *Eimeria mitis*, *Eimeria necatrix*, *Eimeria praecox*, *Eimeria tenella*) are present in all six continents where chickens are found (including 21 countries). Analysis of 248 internal transcribed spacer sequences derived from 17 countries provided evidence of possible allopatric diversity for species such as *E. tenella* (F_ST_ values ⩽0.34) but not *E. acervulina* and *E. mitis*, and highlighted a trend towards widespread genetic variance. We found that three genetic variants described previously only in Australia and southern Africa (operational taxonomic units x, y and z) have a wide distribution across the southern, but not the northern hemisphere. While the drivers for such a polarised distribution of these operational taxonomic unit genotypes remains unclear, the occurrence of genetically variant *Eimeria* may pose a risk to food security and animal welfare in Europe and North America should these parasites spread to the northern hemisphere.

## Highlights

• The seven *Eimeria* spp. recognised to infect chickens are present globally.
• Cryptic *Eimeria* operational taxonomic units (OTUs) are common in the southern but not northern hemisphere.
• Parasite population structure appears to vary between *Eimeria* spp.

## Graphical abstract

**Figure:**
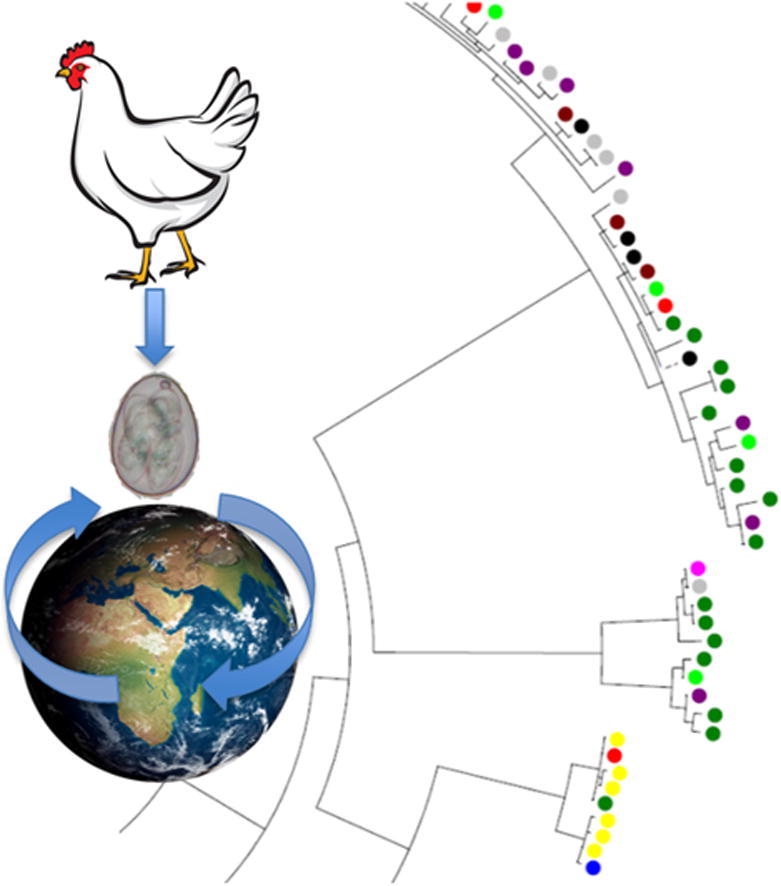

## 1 Introduction

Apicomplexan protozoan parasites of the genus *Eimeria* are obligate intracellular pathogens of huge economic and veterinary importance. Other apicomplexan genera that cause serious human, veterinary or zoonotic diseases include *Neospora*, *Plasmodium*, *Theileria* and *Toxoplasma*. Sustainable food security is a global concern and with the human population set to exceed nine billion by 2050 (O’Neill et al., 2010), demand is mounting for more efficient food production. Chicken is one of the most efficient sources of animal-derived protein (Smil, 2002), thus pathogens that compromise the efficiency of chicken production can pose a serious threat to global food supplies and human poverty (Perry et al., 2002, Godfray et al., 2010). *Eimeria* spp. that cause coccidiosis in domestic chickens (*Gallus gallus domesticus*) have a major impact on animal welfare and the economics of chicken farming (Shirley et al., 2005, Dalloul and Lillehoj, 2006). *Eimeria* spread through chicken houses via the faecal-oral route and conditions for bird-to-bird transmission are highly favoured in the increasingly intensive environments used to rear broiler chickens (Long et al., 1976). Through a combination of parasite ubiquity, fecundity and pathogenicity, coccidiosis is among the 10 most economically significant endemic livestock diseases in the UK and the developed world, and is also one of the top 10 veterinary diseases detrimental to the poor in southern Asia (Perry et al., 2002, Bennett and Ijpelaar, 2005).

The primary means of coccidiosis control is by prophylactic administration of in-feed anticoccidial drugs, although resistance is ubiquitous (Shirley et al., 2007). Vaccination of young (usually day-of-hatch) chicks with live oocyst vaccines comprising non-attenuated (previously wild-type) or attenuated parasites is an effective alternative to drugs, but relative to cost, the requirement for multiple parasite lines and production capacity prove limiting (Shirley et al., 2005). Notwithstanding all of these issues, live vaccines work well when applied in the field and there is to date no substantive evidence that their use over several decades has driven selection of parasite populations towards resistance/immune escape (Shirley et al., 2005). A major reason for their success may well be that live replicating *Eimeria* parasites express between 6000 and 9000 proteins during their developmental life cycle (Reid et al., 2014) and they present the host immune system with a very complex portfolio of antigenic peptides, which may limit opportunities for breakthrough by genetically distinct variants. However, the development and application of next generation subunit or vectored vaccines based on the expression of a single, or a small number, of *Eimeria* antigens could drive more targeted immune selection, leading to the rapid appearance and dissemination of vaccine resistance in the field.

The success of any pathogen control strategy is determined in part by the level of genetic diversity pre-existing in field populations. For *Eimeria,* and other members of the phylum Apicomplexa, assessing such diversity provides an interesting challenge. Cost-effective vaccines and chemoprophylactics are urgently required for all, but naturally occurring genetic polymorphism is proving difficult to overcome. For some apicomplexans such as *Plasmodium* and *Toxoplasma*, our understanding of population structure and the occurrence of genetic diversity is highly detailed and has been studied in depth (e.g. Amambua-Ngwa et al., 2012, Manske et al., 2012, Minot et al., 2012). For *Eimeria* of the chicken there is an extensive literature covering species occurrence and pathognomonic signatures (Shirley et al., 2005, Chapman et al., 2013). Nevertheless, documentation of isolates with uncharacteristic pathogenicity, and the molecular detection of *Eimeria*-specific ribosomal internal transcribed spacer (ITS) DNA sequences that are sufficiently divergent to warrant classification as novel operational taxonomic units (OTUs), indicates that there is an unexplored diversity of these parasites in the field (Cantacessi et al., 2008, Williams et al., 2009, Fornace et al., 2013). Moreover, a recent network analysis of population genetic structure, whole genotype diversity and cross-fertilisation in field samples of a single species, *Eimeria tenella*, indicated considerable geographical variation with some study areas possessing a small number of expanded parasite genotypes, suggestive of limited genetic mixing, whereas others were essentially panmictic (Blake et al., 2015). The potential for parasite population mixing and increased transport of day old chicks facilitated by the opening up of trade routes across the globe may enhance this further.

In this study we have sampled countries from six continents to determine the occurrence of all seven *Eimeria* spp. known to infect the domestic chicken and used genus-specific ITS sequencing to define diversity between and within populations. With sequences from all continents where chickens are raised, this study provides the largest estimate of global diversity of *Eimeria* to date.

## 2 Materials and methods

### 2.1 Ethics statement

This study was carried out in strict accordance with the Animals (Scientific Procedures) Act 1986, an Act of Parliament of the United Kingdom. All sample collection, animal studies and protocols were approved by the Royal Veterinary College (UK) Ethical Review Committee and the United Kingdom Government Home Office under the project licences 30/2545 and 70/7781. All field samples were imported into the UK under Importation of Animal Pathogens Order (IAPO) permits PATH/71/2010/1 and PATH/71/2011/1-3, 6, 9, 10-13 issued by the Department for Environment, Food and Rural Affairs, UK.

### 2.2 Field sample selection

In total 512 faecal samples collected from small to medium scale commercial farms (defined here as holding up to 50,000 broilers or layers) from 20 countries across five continents were tested for the occurrence of *Eimeria* genomic DNA and admitted to the study, supplemented by the addition of one archive isolate collected from Japan (Reid et al., 2014) (Table 1). Data from Australia representing a sixth continent were incorporated using the published literature (Cantacessi et al., 2008, Morgan et al., 2009). Sampling frames were compiled with different approaches in each of the partner countries using records from veterinary services, poultry suppliers and farmer organisations since information, accessibility of farms and legislation governing exportation of biological samples varied. Since the aim of the study was to sample for genetic diversity, farms were chosen to maximise parasite diversity, including intensive and extensive broiler and layer systems. Thus, it should be noted that parasite prevalence has not been determined. The use of chemoprophylaxis varied between farms but no sampled birds had received live anticoccidial vaccination. In countries where 18 or more farms were sampled those identified were compiled into lists of broiler and layer farms. A panel of farms representing each production type in each country was then randomly selected using Microsoft Excel randomiser (Microsoft Corporation, USA). Samples were collected from one pen per farm where birds older than 3 weeks of age were available.

### 2.3 Field sample collection, oocyst recovery and processing

Faecal samples were collected across each poultry unit following a ‘W’ pathway as described previously (Fornace et al., 2013). Oocysts were detected, purified and disrupted for extraction of total genomic DNA using a QIAamp DNA Stool Mini kit (Qiagen, Venlo, Netherlands) as described elsewhere (Kumar et al., 2014).

### 2.4 Nucleic acid resources

Genomic DNA purified from the reference *Eimeria acervulina*, *Eimeria brunetti*, *Eimeria maxima*, *Eimeria mitis*, *Eimeria necatrix*, *Eimeria praecox* and *E. tenella* Houghton strains was used to provide positive control as described previously (Blake et al., 2003, Reid et al., 2014). PCR products were sequenced and those found to represent OTUs x, y or z were cloned as described in Section 2.5 and retained for use as positive controls in subsequent PCRs.

### 2.5 PCR

To avoid the subjectivity associated with microscopic methods (morphometric measurements of oocysts or histopathological appearance of the intestines) and with assessment of gross pathological lesions, we adopted non-quantitative PCR-based screening for *Eimeria* spp. and OTU genotype occurrence. We used primer pairs that have been validated previously for both sensitivity and specificity and for which there is no evidence of primer binding-site polymorphism (Vrba et al., 2010, Fornace et al., 2013, Kumar et al., 2014). PCR amplification was completed using Invitrogen High Fidelity Platinum *Taq* DNA polymerase (Life Technologies, Paisley, UK). Each PCR included 25 ng of template DNA (or molecular grade water for negative control), 1 U High Fidelity *Taq* DNA polymerase, 10× High Fidelity PCR Buffer, 1.5 mM MgSO_4_, 10 mM dNTPs and 20 pmol of the relevant forward and reverse primers. Standard cycle parameters were 1 × 2 min at 94 °C, 30× (30 s at 94 °C, 1 min at 52–60 °C and 1 min per Kb amplicon at 68 °C) and 1 × 10 min at 68 °C. The primers were synthesised by Sigma–Genosys (Haverhill, UK) and are presented, together with the annealing temperatures used, in Supplementary Table S1. PCR fragments were cloned using the Strataclone PCR cloning kit (Agilent, Santa Clara, USA) in Strataclone Solopack Competent Cells (Agilent), miniprepped (Qiagen) and sequenced (GATC Biotech, Konstanz, Germany) as described by the respective manufacturers. Sequence assembly, annotation and interrogation were undertaken using CLC Main Workbench v6.0.2 (CLC Bio, Katrinebjerg, Denmark) except where stated.

The occurrence of *Eimeria* in each field sample was determined by genus-specific PCR targeting the 5S rDNA repeat (Blake et al., 2006). Samples found to contain *Eimeria* DNA were screened for species identification using primers specific for each *Eimeria* sp. and OTU genotype as described previously (Vrba et al., 2010, Fornace et al., 2013). ITS regions 1 and 2 were amplified using genus-specific primers located in conserved regions of the flanking 18S and 28S rRNA genes (Schwarz et al., 2009).

### 2.6 Phylogenetics and sequence analysis

Prior to alignment all ITS1-5.8S-ITS2 sequences were screened using Bellerophon with the Huber-Hugenholtz correction and a 300 bp window to remove any chimeric sequences (Huber et al., 2004). Thirteen chimeric sequences were identified in the first screen, with two more chimeras detected in a second screen of the cleaned data. Tertiary screening revealed no further chimeras. Chimera-free ITS1-5.8S-ITS2 sequences (GenBank accession numbers **LN609768**–**LN609975**) were aligned together with reference sequences downloaded from GenBank using ClustalX through CLC Main Workbench with the ‘very accurate (slow)’ option including a gap open cost of 10 and a gap extension cost of 1 (CLC Bio; downloaded reference accession numbers as shown in Supplementary Table S2). Phylogenetic relatedness was inferred using MEGA5.1 (Tamura et al., 2011) with the maximum likelihood (ML), maximum parsimony (MP) and neighbor joining (NJ) methods. ML was inferred using the Tamura-Nei model with gamma distribution based upon the optimal Akaike Information Criterion (AIC) calculated using jMODELTEST2 (Darriba et al., 2012) with 1000 bootstrap replicates. MP and NJ were also inferred using MEGA5.1, including 1000 bootstrap replicates. Trees were visualised using TreeView v1.6.6 (Page, 1996). Mean distance estimation was calculated for the full ITS dataset, and each species-/OTU-specific subset, using MEGA5.1 and the ML Tamura-Nei model with gamma distribution and 1000 bootstrap replication. Principal co-ordinate analysis was performed for clusters represented by more than 30 sequences (*E. acervulina*, *E. mitis* (short form) and *E. tenella*) based upon pairwise sequence divergence, calculated using maximum composite likelihood with 1000 bootstrap replicates in MEGA5.1. Principal co-ordinate data were visualised using GenAlEx v6.5 (Peakall and Smouse, 2012). ITS1-5.8S-ITS2 sequences were also assessed for species delimitation using bPTP (Zhang et al., 2013), employing a Bayesian Markov Chain Monte Carlo model with 2,000,000 generations, thinning by 200 with a 0.2 burn-in and a seed of 123 in order to achieve convergence. Wrights Fixation Index (F_ST_) was applied in order to assess population structure based upon ITS1-5.8S-ITS2 sequence variation as a result of gene flow between populations using DnaSP v5.10.1. Pairwise fixation values were calculated with 1000 replicates as a permutation test (Hudson et al., 1992, Librado and Rozas, 2009).

## 3 Results

### 3.1 Occurrence of Eimeria spp. that infect chickens and an expanded geographic range for genetic variants

**Fig. 1:**
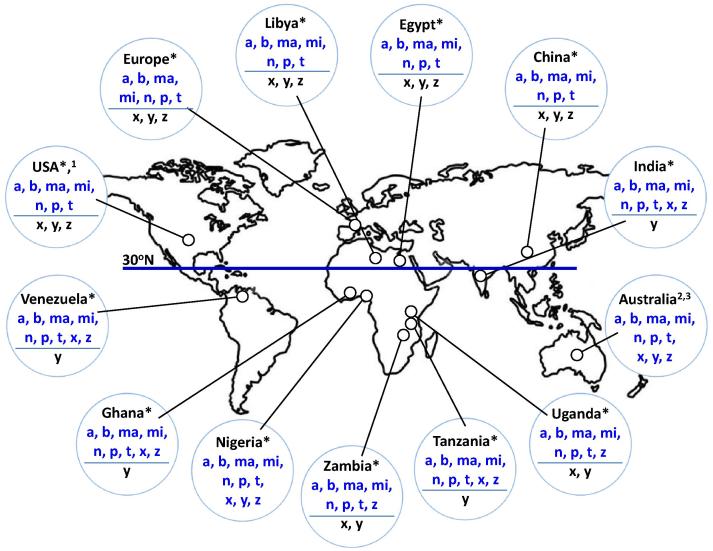
Assembling a global panel of *Eimeria* field samples: countries sampled and parasite occurrence determined by species-specific PCR. Twenty-one countries were sampled for *Eimeria* spp. occurrence, including all six continents where domestic chickens can be found. The number of samples used for species detection by PCR in each country and the results per species are shown in Table 1. The line labelled 30°N indicates 30° latitude north. Species or operational taxonomic units (OTUs) found to be present in each country/region are shown in blue above the line in each circle, OTUs found to be absent are shown in black beneath the line. a, *Eimeria acervulina*; b, *Eimeria brunetti*; ma, *Eimeria maxima*; mi, *Eimeria mitis*; n, *Eimeria necatrix*; p, E*imeria praecox*; t, *Eimeria tenella*; x, OTUx; y, OTUy; z, OTUz. ^∗^Data produced in this study. Superscript numbers indicate data derived from the published literature ^1^Schwarz et al. (2009); ^2^Cantacessi et al. (2008); ^3^Morgan et al. (2009).

Regional studies of *Eimeria* spp. occurrence are plentiful, although variation in the methods used for species identification makes direct comparison difficult (Haug et al., 2008, Kumar et al., 2014). Here, faecal samples were collected from 512 poultry units across 20 countries, representing five continents (Table 1). Adding published molecular data from Australia, China and the United States (US), we identified all seven *Eimeria* spp. known to infect domestic chickens across each of the six continents where chickens are raised (Fig. 1, Table 1 and Supplementary Table S2) (Cantacessi et al., 2008, Schwarz et al., 2009, Reid et al., 2014). Extension of the PCR survey to include sequences which define OTUs x, y and z identified at least one OTU variant in every country sampled south of 30°N latitude. In contrast no OTU variant was identified north of this latitude, despite extensive sampling. The OTUz variant was most common, appearing in all eight countries represented south of 30^o^N where it was detected in up to a third of all flocks sampled (Table 1). The OTUx genotype was similarly common, being detected in six of eight countries south of 30^o^N and found in up to a quarter of all samples. OTUy was detected only in Nigeria, the most extensively sampled country south of 30°N.

### 3.2 Comparison of Eimeria ITS sequences indicate varied levels of diversity among countries and continents

**Fig. 2:**
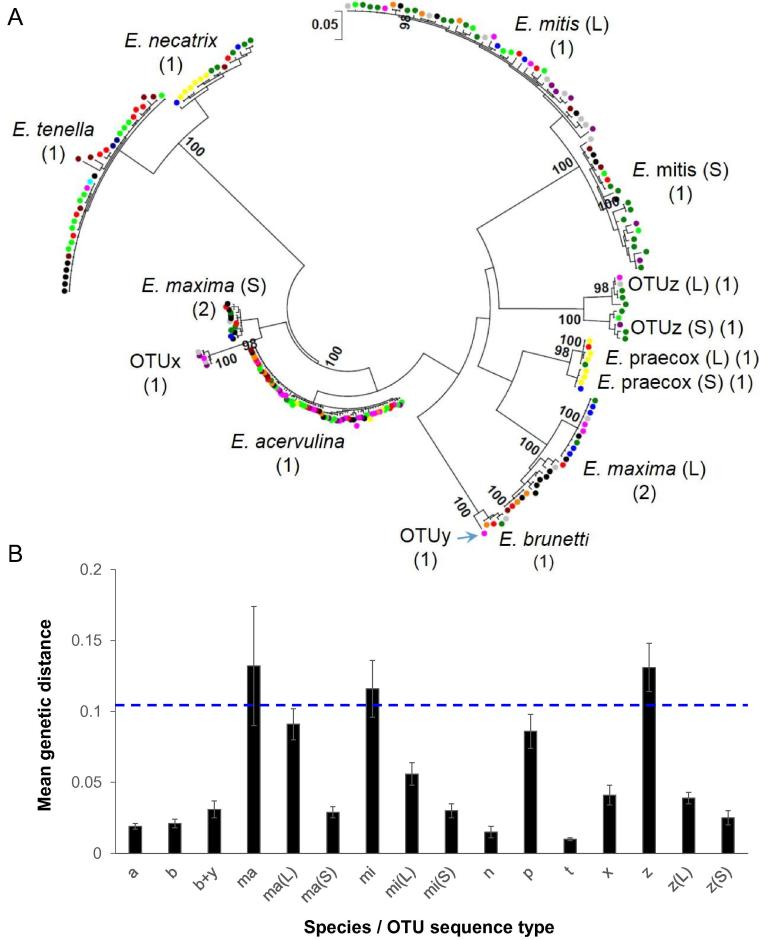
*Eimeria* spp. and operational taxonomic unit (OTU) internal transcribed spacer sequence (ITS) 1 and 2 diversity. (A) Tamura-Nei model Maximum Likelihood (ML) phylogeny of ITS1-5.8S-ITS2 sequences derived with 1000 bootstrap replication from samples collected in Asia, Africa, Europe and the Americas (GenBank accession numbers **LN609768**–**LN609975**). The number and origin of sequences used are shown in Table 1. Coloured spots indicate the country of origin for each sequence (Asia: China (blue), India (dark blue), Japan, (light blue). Europe: countries pooled (red). North Africa: Egypt (orange), Libya (dark red). Sub-Saharan Africa: Ghana (purple), Nigeria (pink), Tanzania (dark green), Uganda (green), Zambia (yellow). Americas: USA (black), Venezuela (grey)). L, long sequence form; S, short sequence form. Numbers shown in parentheses indicate the number of putative species partitioned per recognised species, sequence form or OTU by Bayesian species delimitation. (B) Mean genetic distance within each species and OTU genotype calculated using ML with 1000 bootstrap replications. Species and genotype identifiers are as follows: a, *Eimeria acervulina*; b, *Eimeria brunetti*; ma, *Eimeria maxima*; mi, *Eimeria mitis*; n, *Eimeria necatrix*; p, E*imeria praecox*; t, *Eimeria tenella*; x, OTUx; y, OTUy; z, OTUz; L, long sequence form; S, short sequence form. The dotted line indicates the intersect between the combined and the separated long and short sequence forms.

Sequence-led studies of genetic diversity within *Eimeria* spp. have most commonly focused on sequences that extend across the ITS regions between the rDNA genes (Beck et al., 2009). The large number of copies of this cluster per genome provides a high degree of sensitivity by PCR, although the occurrence of polymorphism between copies, even within single genomes, can result in overestimation of clonal diversity. Nonetheless, ITS remains the most widely sequenced locus for *Eimeria* and the only way in which the OTU genotypes may be detected. In total 207 new ITS1-5.8S-ITS2 sequences were produced from samples derived from 15 countries across five continents and were combined with 43 published sequences from China and the USA (GenBank accession numbers **LN609768**–**LN609975**, Table 1, plus the published sequences shown in Supplementary Table S2). Sequences defining Australian OTU ITS1 sequences were not previously available. ML phylogenetic comparison using the Tamura-Nei model, with a gamma distribution and 1000 bootstrap replicates, reproducibly resolved clades for *E. acervulina*, *E. brunetti*, *E. necatrix*, *E. praecox* and *E. tenella*, with distinct branches for the long and short form sequences of *E. maxima* and *E. mitis* as described previously (Schwarz et al., 2009) (Fig. 2A). Sequences annotated as OTUx by comparative similarity of the ITS2 sequence component with sequences previously deposited in GenBank (Cantacessi et al., 2008) were found to cluster alongside the *E. maxima* short form. A single sequence defined as OTUy was detected and found to cluster with *E. brunetti*, with sufficient similarity to be considered the same species. Sequences annotated as OTUz formed a distinct clade with clear sub-branches and high bootstrap support, including subdivision of long and short sequence forms (Fig. 2A). Comparable results were achieved using MP and NJ, although topology within species/sequence length/taxonomic unit forms was inconsistent. Mean overall genetic distance was calculated using ML with 1000 bootstrap replicates to be 0.46 ± 0.09. Mean overall genetic distance per species ranged from 0.01 ± 0.004 to 0.13 ± 0.04, although separation of the *E. maxima* and *E. mitis* long and short forms brought the maximum down to 0.09 ± 0.01 (Fig. 2B). The mean overall genetic distance calculated for OTUz was 0.13 ± 0.02, which reduced to 0.04 ± 0.004 and 0.03 ± 0.005 when separated by sequence length form. Including the single OTUy sequence with *E. brunetti* increased genetic distance by just 0.01, from 0.02 ± 0.003 to 0.03 ± 0.006.

Using bPTP to calculate species delimitation for the ITS1-5.8S-ITS2 sequences resulted in an overprediction of possible species (Fig. 2A). Sequences representing *E. acervulina*, *E. brunetti*, *E. necatrix* and *E. tenella* were partitioned as discrete species. *Eimeria maxima* and *E. mitis* partitioned into long and short sequence types, although both *E. maxima* forms presented further subdivisions. Unexpectedly, *E. praecox* also partitioned into two groups represented by long and short sequence forms (1084/1092-1139/1150 bp, respectively). All three OTU types partitioned separately, with distinct long and short sequence types for OTUz.

**Fig. 3:**
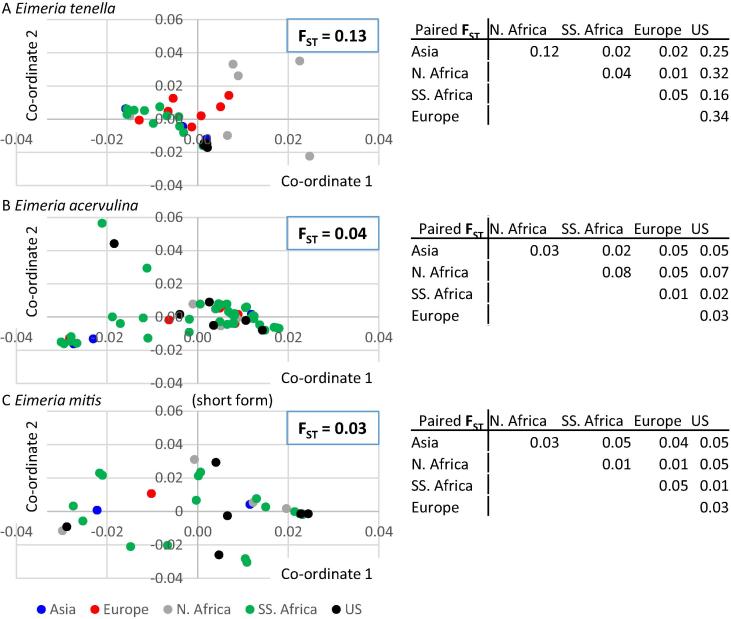
Internal transcribed spacer (ITS) 1-5.8S-ITS2 sequence principal co-ordinate analysis and Wrights Fixation Index (F_ST_), illustrating variation for (A) *Eimeria tenella* in some regions, but not (B) *Eimeria acervulina* or (C) *Eimeria mitis*. N. Africa, North Africa; SS. Africa, sub-Saharan Africa. Pairwise and overall F_ST_ values are shown beside the equivalent plot.

To define the extent of variation in sequence diversity we calculated the F_ST_ using ITS1-5.8S-ITS2 for those *Eimeria* spp. represented by more than 30 sequences (*n* = 3), subdivided as Asia, Europe, North Africa, Sub-Saharan Africa and the USA. Average F_ST_ of 0.04, 0.03 and 0.13 (*E. acervulina*, *E. mitis* – short form and *E. tenella*) suggested species-specific variation in levels of interbreeding. Pairwise analysis revealed overlapping genetic distance between spatially distinct *E. acervulina* and *E. mitis* populations, with a conflictingly higher level of genetic isolation between some *E. tenella* populations (Fig. 3). Principal co-ordinate analysis was performed separately for these three *Eimeria* spp. based upon pairwise sequence divergence, calculated using maximum composite likelihood with 1000 bootstrap replicates and illustrating distinct variation in diversity between some countries or continents for *E. tenella* but not *E. acervulina* or *E. mitis* (Fig. 3).

## 4 Discussion

Regional surveys of *Eimeria* spp. in chickens have commonly identified the presence of multiple species of parasite but have often suggested that one or more species are absent from particular geographic regions (e.g. (Al-Natour et al., 2002, Haug et al., 2008, Hamidinejat et al., 2010, Gyorke et al., 2013)). Such variation in species detection may be attributable to species-specific differences in abundance or to indistinct pathognomonic signatures, hindering microscopic, molecular or pathological detection. Here, analysis of a large panel of *Eimeria* genomic DNAs extracted from faeces collected from chicken farms around the world has shown the occurrence of all seven recognised *Eimeria* spp. in every country and region tested. Phylogenetic analysis of the ITS1-5.8S-ITS2 locus of these samples revealed varied levels of diversity for *E. tenella* between countries and continents, in line with recent reports of genome-wide haplotype diversity (Blake et al., 2015). Comparable variation in diversity was not detected for *E. acervulina* or *E. mitis*. While we have not assessed the prevalence of each *Eimeria* sp. within any of the countries and regions tested, it is clear that complete control of coccidiosis will require the targeting of all seven species. Of particular interest, this survey has identified that the cryptic *Eimeria* genotypes termed OTUx, y and z have a much wider and apparently more geographically polarised occurrence than previously described, with striking variation between northern and southern hemispheres. Originally described in parasite DNA extracted from chickens in Australia (Morris et al., 2007, Cantacessi et al., 2008), all three genotypes are now common in the region with reports of occurrence and transmission identifying the chicken as a true host (Morris et al., 2007, Godwin and Morgan, 2014, Godwin and Morgan, 2015). OTUx and OTUz sequences were also recently detected in parasite DNA from Ghana, Tanzania and Zambia (Fornace et al., 2013). We have now found OTUx and OTUz genotypes dispersed throughout southern Asia, Sub-Saharan Africa and at least one South American country at levels comparable with species such as *E. maxima* and *E. necatrix*, but to date we have not detected them north of 30^o^N latitude, despite large-scale sampling and scrutiny of published genus-specific primed ITS2 sequence resources (Supplementary Table S2). Furthermore, we have now detected the OTUy genotype outside of Australia in samples collected from commercial chicken farms in Nigeria. The close genetic relationship between OTUy and *E. brunetti* suggests the former should be identified as *E. brunetti* pending further genomic and parasitological analysis. Similarly, OTUx may be a divergent *E. maxima* lineage. OTUz appears most distinct, including long and short sequence types as described for *E. maxima* and *E. mitis*, and now *E. praecox*, and is considered most likely to be a new species. We applied Bayesian species delimitation in an attempt to resolve those relationships between *Eimeria* spp. and OTUs. While all three OTUs did partition separately, comparable partitioning within three recognised species preclude confident taxonomic assignment. Analysis of additional, more stable loci will be required (Ogedengbe et al., 2011).

The factor(s) underlying the apparent polarised distribution of OTU genotypes around the world are not known. Indigenous chickens do present distinct region-specific host haplotypes across much of the world and susceptibility to *Eimeria* is known to vary by host genotype (Bumstead and Millard, 1992, Dancause et al., 2011, Umaya Suganthi, 2014), but indigenous birds are overwhelmingly in the minority in all of the regions sampled and were not sampled in Nigeria. The dominant genotypes of chickens in layer and broiler farms throughout the world are supplied to farmers by a small number of globally active companies. Similarly, comparable chemoprophylactic strategies employing a common panel of anticoccidial drugs were recorded in each country. Trade routes used for the distribution of commercial broiler-breeders (the parents and grand-parents of birds reared for their meat) could provide another possible explanation for OTU parasite dissemination, although current routes are not consistent with the observed distribution (Emsley, 2006) and parasite transmission by this means is very unlikely given the acute nature of infection and lack of persistence within the host. In evolutionary terms commercial chicken genotypes are recent introductions to much of the southern hemisphere and the indigenous chicken breeds that they have largely replaced, but have also interbred with, are highly varied across the regions found to contain OTU genotype parasites (Umaya Suganthi, 2014). We have shown that the occurrence of the seven recognised *Eimeria* spp. that infect chickens is not affected by location in the same way as the OTUs, indicating that geographic location alone is an unlikely explanation for the polarised distribution. Discovery of the OTU genotypes within the last 10 years might indicate a recent emergence, in which case it is plausible that these variant parasites may be migrating through a combination of local bird and staff movement and mechanical transmission. Their emergence might be a consequence of genetic drift through geographic isolation, although host switching from another galliform(s) with a more regional distribution might also be considered.

Comparison of Australian ITS2 sequences provided the first, and until now only, locus definition of the *Eimeria* OTU genotypes (Cantacessi et al., 2008). ITS sequencing here supported definition of the OTU genotype range and identification of ITS1 sequences for OTUs x, y and z. A clear direction for future work is to address our understanding of the population structure of all *Eimeria* spp. in the field as reported recently for *E. tenella* (Blake et al., 2015). Genetic analysis of the ITS1-5.8S-ITS2 sequences generated here and retrieved from public databases revealed notable variation in population structure. *Eimeria acervulina* and *E. mitis* present signatures indicative of regular interbreeding between genotypes. In contrast, higher F_ST_ figures for *E. tenella* suggest a more restricted population structure in line with recent genome-wide single nucleotide polymorphism (SNP) haplotype-based analyses (Blake et al., 2015). Underlying factors for such variation between species may include a faster generation time and greater fecundity for *E. acervulina* and *E. mitis* compared with *E. tenella* (∼33% shorter prepatent period and 2.5–4× the oocyst output per oocyst ingested (Bumstead and Millard, 1992, Eckert et al., 1995)); providing the former species greater opportunity for co-infection, hybridisation and genome evolution. Other explanations might include a more recent emergence, possibly following a switch from another galliform host. Recent phylogenetic analysis using 18S rDNA and cytochrome oxidase subunit I sequences have indicated a closer relationship for *E. tenella* and *E. necatrix* to *Eimeria* from turkeys than other *Eimeria* from chickens (Miska et al., 2010, Vrba and Pakandl, 2015). Reports of immune responses induced in chickens following *Eimeria adenoides* infection which provide partial immune protection against subsequent *E. tenella* challenge offer further support (Augustine and Danforth, 1990), although host specificity has been confirmed for *E. tenella* (Vrba and Pakandl, 2015). Confirmation of these findings will require analysis of additional loci. Genetic mapping has already been used extensively as a technique to investigate genetic differences in the coccidia (reviewed in Clark and Blake (2012)). Now, the rapidly falling cost of next-generation sequencing technologies means that high depth whole genome population genetic analysis of field populations is starting to become feasible for these parasites.

How genetic, particularly antigenic, diversity influences pathogenicity and epidemiology and the implications of this for effective intervention and control are important questions for all apicomplexan parasites. The widespread occurrence of genetically divergent strains, and possibly even species, of *Eimeria* that may be capable of replicating within chickens vaccinated using current generation vaccines indicates a significant risk to food security and animal welfare should these parasites spread to the northern hemisphere.

## Figures and Tables

**Table 1:** Parasite occurrence and geographic origin of samples and sequences used during the current studies. Data are indicative of occurrence not prevalence. Small numbers of samples were collected from Belgium, France, Germany, Ireland, Italy, Poland, Portugal, Spain and the United Kingdom and grouped together here as Europe, with those from the countries shown underlined here used to generate sequences. European data were combined to provide a statistically significant unit.

Country	Farms sampled for species detection by PCR (No. negative)	ITS1-5.8S rDNA-ITS2 sequences used^a^ (No. samples used for PCR)	No. farms positive for *Eimeria* spp./genotype occurrence (%)									
*E. acervulina*	*E. brunetti*	*E. maxima*	*E. mitis*	*E. necatrix*	*E. praecox*	*E. tenella*	OTUx	OTUy	OTUz
Egypt	44 (12)	12 (5)	16 (36.4)	5 (11.4)	14 (31.8)	9 (20.5)	1 (2.3)	2 (4.5)	7 (15.9)	0 (0.0)	0 (0.0)	0 (0.0)
Libya	62 (22)	16 (5)	13 (21.0)	1 (1.6)	8 (12.9)	9 (14.5)	14 (22.6)	2 (3.2)	18 (29.0)	0 (0.0)	0 (0.0)	0 (0.0)
Ghana	18 (0)	16 (5)	12 (66.7)	1 (5.6)	2 (11.1)	7 (38.9)	3 (16.7)	6 (33.3)	7 (38.9)	3 (16.7)	0 (0.0)	2 (11.1)
Nigeria	59 (5)	30 (12)	26 (44.1)	4 (6.8)	9 (15.3)	16 (27.1)	7 (11.9)	2 (3.4)	35 (59.3)	10 (16.9)	2 (3.4)	9 (15.3)
Tanzania	38 (1)	39 (12)	22 (57.9)	5 (13.2)	7 (18.4)	12 (31.6)	7 (18.4)	15 (39.5)	19 (50.0)	9 (23.7)	0 (0.0)	7 (18.4)
Uganda	6 (0)	35 (5)	4 (66.7)	2 (33.3)	1 (16.7)	1 (16.7)	2 (33.3)	1 (16.7)	3 (50.0)	1 (16.7)	0 (0.0)	2 (33.3)
Zambia	40 (9)	15 (5)	20 (50.0)	1 (2.5)	3 (7.5)	2 (5.0)	8 (20.0)	8 (20.0)	9 (22.5)	2 (5.0)	0 (0.0)	1 (2.5)
India	198 (47)	2 (2)	65 (32.8)	7 (3.5)	32 (16.2)	56 (28.3)	44 (22.2)	32 (16.2)	122 (61.6)	3 (1.5)	0 (0.0)	11 (5.6)
Japan	0	1 (1)	nd	nd	nd	nd	nd	nd	nd	nd	nd	nd
China	11 (NR)	11^b^	4 (36.4)	3 (27.3)	2 (18.2)	2 (18.2)	1 (9.1)	3 (27.3)	7 (63.6)	0 (0.0)	0 (0.0)	0 (0.0)
Europe	16 (NR)	24 (5)	7 (20.6)	1 (2.9)	2 (5.9)	4 (11.8)	1 (2.9)	2 (5.9)	6 (17.6)	0 (0.0)	0 (0.0)	0 (0.0)
USA	16 (NR)	1+31^b^	11 (68.8)	1 (6.3)	3 (18.8)	1 (6.3)	1 (6.3)	2 (12.5)	6 (37.5)	0 (0.0)	0 (0.0)	0 (0.0)
Venezuela	4 (0)	15 (4)	4 (100.0)	1 (25.0)	1 (25.0)	2 (50.0)	1 (25.0)	1 (25.0)	3 (75.0)	1 (25.0)	0 (0.0)	1 (25.0)

Total: 21	512	248										

nd, not done; NR, not relevant; OTU, Operational Taxonomic Units; ITS, internal transcribed spacer.

a The published sequences used are available in Supplementary Table S2.

b GenBank accession numbers **LN609768**–**LN609975**.

## Appendix A Supplementary data

Supplementary Table S1 Details of primers used in this study, their source, annealing temperature and expected amplicon size.

Supplementary Table S2 Summary and GenBank accession numbers of published internal transcribed spacer (ITS) with/without 5.8S rDNA sequences used during these studies. Sequences represent *Eimeria acervulina*, *Eimeria brunetti*, *Eimeria maxima*, *Eimeria necatrix*, *Eimeria praecox* and *Eimeria tenella*, as well as operational taxonomic units (OTUs) x, y and z.
