# Supplementary material for: Cryptic Eimeria genotypes are common across the southern but not northern hemisphere
Source: Int J Parasitol. 2016 Aug;46(9):537–44. doi: 10.1016/j.ijpara.2016.05.006 (PMC4978698; doi:10.1016/j.ijpara.2016.05.006)
Supplement: Supplementary Table S1 — Details of primers used in this study, their source, annealing temperature and expected amplicon size. [file mmc1.docx]

**Supplementary Table S1.** Details of primers used in this study, their source, annealing temperature and expected amplicon size.

| **Target** | **Name** | **Sequence (5' - 3')** | **Source** | **Annealing temperature** | **Amplicon size (bp)** |
| --- | --- | --- | --- | --- | --- |
| *Eimeria acervulina* | ACE-F | GCAGTCCGATGAAAGGTATTTG | Vrba et al., 2010 | 56 ^o^C | 103 |
| (diagnostic) | ACE-R | GAAGCGAAATGTTAGGCCATCT | Vrba et al., 2010 |  |  |
| *Eimeria brunetti* | BRU-F | AGCGTGTAATCTGCTTTTGGAA | Vrba et al., 2010 | 56 ^o^C | 118 |
| (diagnostic) | BRU-R | TGGTCGCAGACGTATATTAGGG | Vrba et al., 2010 |  |  |
| *Eimeria maxima* | MAX-F | TCGTTGCATTCGACAGATTC | Vrba et al., 2010 | 56 ^o^C | 138 |
| (diagnostic) | MAX-R | TAGCGACTGCTCAAGGGTTT | Vrba et al., 2010 |  |  |
| *Eimeria mitis* | MIT-F | CAAGGGGATGCATGGAATATAA | Vrba et al., 2010 | 56 ^o^C | 115 |
| (diagnostic) | MIT-R | CAAGACGAATGGAATCAATCTG | Vrba et al., 2010 |  |  |
| *Eimeria necatrix* | NEC-F | AACGCCGGTATGCCTCGTCG | Vrba et al., 2010 | 56 ^o^C | 134 |
| (diagnostic) | NEC-R | GTACTGGTGCCAACGGAGA | Vrba et al., 2010 |  |  |
| *Eimeria praecox* | PRA-F | CACATCCAATGCGATATAGGG | Vrba et al., 2010 | 56 ^o^C | 117 |
| (diagnostic) | PRA-R | ACAGAAAAACGCAAAGAGCAA | Vrba et al., 2010 |  |  |
| *Eimeria tenella* | TEN-F | TCGTCTTTGGCTGGCTATTC | Vrba et al., 2010 | 56 ^o^C | 100 |
| (diagnostic) | TEN-R | CAGAGAGTCGCCGTCACAGT | Vrba et al., 2010 |  |  |
| OTUx | OTU_X_f1 | GTGGTGTCGTCTGCGCGT | Fornace et al., 2013 | 56 ^o^C | 133 |
| (diagnostic) | OTU_X_r1 | ACCACCGTATCTCTTTCGTGA | Fornace et al., 2013 |  |  |
| OTUy | OTU_Y_f1 | CAAGAAGTACACTACCACAGCATG | Fornace et al., 2013 | 56 ^o^C | 346 |
| (diagnostic) | OTU_Y_r1 | ACTGATTTCAGGTCTAAAACGAAT | Fornace et al., 2013 |  |  |
| OTUz | OTU_Z_f1 | TATAGTTTCTTTTGCGCGTTGC | Fornace et al., 2013 | 56 ^o^C | 147 |
| (diagnostic) | OTU_Z_r1 | CATATCTCTTTCATGAACGAAAGG | Fornace et al., 2013 |  |  |
| *Eimeria* 5S rRNA | 5S_For | TCATCACCCAAAGGGATT | Blake et al., 2006 | 56 ^o^C | ~110 |
|  | 5S_Rev | TTCATACTGCGTCTAATGCAC | Blake et al., 2006 |  |  |
| *Eimeria* ITS 1 & 2 | ITS_1 | GGATGCAAAAGTCGTAACACGG | Schwarz et al., 2009 | 52 ^o^C | ~873 - ~1,010 |
|  | ITS_2 | TCCTCCGCTTAATAATATGC | Schwarz et al., 2009 |  |  |

OTU, Operational Taxonomic Unit.

**References**

Blake, D.P., Hesketh, P., Archer, A., Shirley, M.W., Smith, A.L., 2006. *Eimeria maxima*: the influence of host genotype on parasite reproduction as revealed by quantitative real-time PCR. Int J Parasitol 36, 97-105.

Fornace, K.M., Clark, E.L., Macdonald, S.E., Namangala, B., Karimuribo, E., Awuni, J.A., Thieme, O., Blake, D.P., Rushton, J., 2013. Occurrence of *Eimeria* species parasites on small-scale commercial chicken farms in Africa and indication of economic profitability. PLoS ONE 8, e84254.

Schwarz, R.S., Jenkins, M.C., Klopp, S., Miska, K.B., 2009. Genomic analysis of *Eimeria* spp. populations in relation to performance levels of broiler chicken farms in Arkansas and North Carolina. J Parasitol 95, 871-880.

Vrba, V., Blake, D.P., Poplstein, M., 2010. Quantitative real-time PCR assays for detection and quantification of all seven *Eimeria* species that infect the chicken. Vet Parasitol 174, 183-190.
