# Supplementary material for: Cryptic Eimeria genotypes are common across the southern but not northern hemisphere
Source: Int J Parasitol. 2016 Aug;46(9):537–44. doi: 10.1016/j.ijpara.2016.05.006 (PMC4978698; doi:10.1016/j.ijpara.2016.05.006)
Supplement: Supplementary Table S2 — Summary and GenBank accession numbers of published internal transcribed spacer (ITS) with/without 5.8S rDNA sequences used during these studies. Sequences represent Eimeria acervulina, Eimeria brunetti, Eimeria maxima, Eimeria necatrix, Eimeria praecox and Eimeria tenella, as well as operational taxonomic units (OTUs) x, y and z. [file mmc2.docx]

**Supplementary Table S2.** Summary and GenBank accession numbers of published internal transcribed spacer (ITS) with/without 5.8S rDNA sequences used during these studies. Sequences represent *Eimeria acervulina*, *Eimeria brunetti*, *Eimeria maxima*, *Eimeria necatrix*, *Eimeria praecox* and *Eimeria tenella*, as well as operational taxonomic units (OTUs) x, y and z.

| **Accession number** | **Locus** | **Country of origin** |
| --- | --- | --- |
| AF027722.1 | ITS2 | USA |
| AF027723.1 | ITS2 | USA |
| AF027724.1 | ITS2 | USA |
| AF027725.1 | ITS2 | USA |
| AF027726.1 | ITS2 | USA |
| AM922227.1 | ITS2 | Australia |
| AM922228.1 | ITS2 | Australia |
| AM922229.1 | ITS2 | Australia |
| AM922230.1 | ITS2 | Australia |
| AM922231.1 | ITS2 | Australia |
| AM922232.1 | ITS2 | Australia |
| AM922233.1 | ITS2 | Australia |
| AM922234.1 | ITS2 | Australia |
| AM922235.1 | ITS2 | Australia |
| AM922236.1 | ITS2 | Australia |
| AM922237.1 | ITS2 | Australia |
| AM922238.1 | ITS2 | Australia |
| AM922239.1 | ITS2 | Australia |
| AM922240.1 | ITS2 | Australia |
| AM922241.1 | ITS2 | Australia |
| AM922242.1 | ITS2 | Australia |
| AM922243.1 | ITS2 | Australia |
| AM922244.1 | ITS2 | Australia |
| AM922245.1 | ITS2 | Australia |
| AM922246.1 | ITS2 | Australia |
| AM922247.1 | ITS2 | Australia |
| AM922248.1 | ITS2 | Australia |
| AM922249.1 | ITS2 | Australia |
| AM922250.1 | ITS2 | Australia |
| AM922251.1 | ITS2 | Australia |
| AM922252.1 | ITS2 | Australia |
| AM922253.1 | ITS2 | Australia |
| AM922254.1 | ITS2 | Australia |
| AM922255.1 | ITS2 | Australia |
| AM922256.1 | ITS2 | Australia |
| AM922257.1 | ITS2 | Australia |
| AM922258.1 | ITS2 | Australia |
| AY779385.1 | ITS2 | USA |
| AY779386.1 | ITS2 | USA |
| AY779387.1 | ITS2 | USA |
| AY779388.1 | ITS2 | USA |
| AY779389.1 | ITS2 | USA |
| AY779390.1 | ITS2 | USA |
| AY779391.1 | ITS2 | USA |
| AY779392.1 | ITS2 | USA |
| AY779393.1 | ITS2 | USA |
| AY779394.1 | ITS2 | USA |
| AY779395.1 | ITS2 | USA |
| AY779396.1 | ITS2 | USA |
| AY779397.1 | ITS2 | USA |
| AY779398.1 | ITS2 | USA |
| AY779399.1 | ITS2 | USA |
| AY779400.1 | ITS2 | USA |
| AY779401.1 | ITS2 | USA |
| AY779402.1 | ITS2 | USA |
| AY779403.1 | ITS2 | USA |
| AY779404.1 | ITS2 | USA |
| AY779405.1 | ITS2 | USA |
| AY779406.1 | ITS2 | USA |
| AY779407.1 | ITS2 | USA |
| AY779408.1 | ITS2 | USA |
| AY779409.1 | ITS2 | USA |
| AY779410.1 | ITS2 | USA |
| AY779411.1 | ITS2 | USA |
| AY779412.1 | ITS2 | USA |
| AY779413.1 | ITS2 | USA |
| AY779414.1 | ITS2 | USA |
| AY779415.1 | ITS2 | USA |
| AY779416.1 | ITS2 | USA |
| AY779417.1 | ITS2 | USA |
| AY779418.1 | ITS2 | USA |
| AY779419.1 | ITS2 | USA |
| AY779420.1 | ITS2 | USA |
| AY779421.1 | ITS2 | USA |
| AY779422.1 | ITS2 | USA |
| AY779423.1 | ITS2 | USA |
| AY779424.1 | ITS2 | USA |
| AY779425.1 | ITS2 | USA |
| AY779426.1 | ITS2 | USA |
| AY779427.1 | ITS2 | USA |
| AY779428.1 | ITS2 | USA |
| AY779429.1 | ITS2 | USA |
| AY779430.1 | ITS2 | USA |
| AY779431.1 | ITS2 | USA |
| AY779432.1 | ITS2 | USA |
| AY779433.1 | ITS2 | USA |
| AY779434.1 | ITS2 | USA |
| AY779435.1 | ITS2 | USA |
| AY779436.1 | ITS2 | USA |
| AY779437.1 | ITS2 | USA |
| AY779438.1 | ITS2 | USA |
| AY779439.1 | ITS2 | USA |
| AY779440.1 | ITS2 | USA |
| AY779441.1 | ITS2 | USA |
| AY779442.1 | ITS2 | USA |
| AY779443.1 | ITS2 | USA |
| AY779444.1 | ITS2 | USA |
| AY779445.1 | ITS2 | USA |
| AY779446.1 | ITS2 | USA |
| AY779447.1 | ITS2 | USA |
| AY779448.1 | ITS2 | USA |
| AY779449.1 | ITS2 | USA |
| AY779450.1 | ITS2 | USA |
| AY779451.1 | ITS2 | USA |
| AY779452.1 | ITS2 | USA |
| AY779453.1 | ITS2 | USA |
| AY779454.1 | ITS2 | USA |
| AY779455.1 | ITS2 | USA |
| AY779456.1 | ITS2 | USA |
| AY779457.1 | ITS2 | USA |
| AY779458.1 | ITS2 | USA |
| AY779459.1 | ITS2 | USA |
| AY779460.1 | ITS2 | USA |
| AY779461.1 | ITS2 | USA |
| AY779462.1 | ITS2 | USA |
| AY779463.1 | ITS2 | USA |
| AY779464.1 | ITS2 | USA |
| AY779465.1 | ITS2 | USA |
| AY779466.1 | ITS2 | USA |
| AY779467.1 | ITS2 | USA |
| AY779468.1 | ITS2 | USA |
| AY779469.1 | ITS2 | USA |
| AY779470.1 | ITS2 | USA |
| AY779471.1 | ITS2 | USA |
| AY779472.1 | ITS2 | USA |
| AY779473.1 | ITS2 | USA |
| AY779474.1 | ITS2 | USA |
| AY779475.1 | ITS2 | USA |
| AY779476.1 | ITS2 | USA |
| AY779477.1 | ITS2 | USA |
| AY779478.1 | ITS2 | USA |
| AY779479.1 | ITS2 | USA |
| AY779480.1 | ITS2 | USA |
| AY779481.1 | ITS2 | USA |
| AY779482.1 | ITS2 | USA |
| AY779483.1 | ITS2 | USA |
| AY779484.1 | ITS2 | USA |
| AY779485.1 | ITS2 | USA |
| AY779486.1 | ITS2 | USA |
| AY779487.1 | ITS2 | USA |
| AY779488.1 | ITS2 | USA |
| AY779489.1 | ITS2 | USA |
| AY779490.1 | ITS2 | USA |
| AY779491.1 | ITS2 | USA |
| AY779492.1 | ITS2 | USA |
| AY779504.1 | ITS2 | USA |
| AY779505.1 | ITS2 | USA |
| AY779506.1 | ITS2 | USA |
| AY779507.1 | ITS2 | USA |
| AY779508.1 | ITS2 | USA |
| AY779509.1 | ITS2 | USA |
| AY779510.1 | ITS2 | USA |
| AY779511.1 | ITS2 | USA |
| AY779512.1 | ITS2 | USA |
| AY779513.1 | ITS2 | USA |
| AY779514.1 | ITS2 | USA |
| FJ230311.1 | ITS2 | USA |
| FJ230312.1 | ITS2 | USA |
| FJ230313.1 | ITS2 | USA |
| FJ230314.1 | ITS2 | USA |
| FJ230315.1 | ITS2 | USA |
| FJ230316.1 | ITS2 | USA |
| FJ230317.1 | ITS2 | USA |
| FJ230318.1 | ITS2 | USA |
| FJ230319.1 | ITS2 | USA |
| FJ230320.1 | ITS2 | USA |
| FJ230321.1 | ITS2 | USA |
| FJ230322.1 | ITS2 | USA |
| FJ230323.1 | ITS2 | USA |
| FJ230324.1 | ITS2 | USA |
| FJ230325.1 | ITS2 | USA |
| FJ230326.1 | ITS2 | USA |
| FJ230327.1 | ITS2 | USA |
| FJ230328.1 | ITS2 | USA |
| FJ230329.1 | ITS2 | USA |
| FJ230330.1 | ITS2 | USA |
| FJ230331.1 | ITS2 | USA |
| FJ230332.1 | ITS2 | USA |
| FJ230333.1 | ITS2 | USA |
| FJ230334.1 | ITS2 | USA |
| FJ230335.1 | ITS2 | USA |
| FJ230336.1 | ITS2 | USA |
| FJ230337.1 | ITS2 | USA |
| FJ230338.1 | ITS2 | USA |
| FJ230339.1 | ITS2 | USA |
| FJ230340.1 | ITS2 | USA |
| FJ230341.1 | ITS2 | USA |
| FJ230342.1 | ITS2 | USA |
| FJ230343.1 | ITS2 | USA |
| FJ230344.1 | ITS2 | USA |
| FJ230345.1 | ITS2 | USA |
| FJ230346.1 | ITS2 | USA |
| FJ230347.1 | ITS2 | USA |
| FJ230348.1 | ITS2 | USA |
| FJ230349.1 | ITS2 | USA |
| FJ230350.1 | ITS2 | USA |
| FJ230351.1 | ITS2 | USA |
| FJ230352.1 | ITS2 | USA |
| FJ230353.1 | ITS2 | USA |
| FJ230354.1 | ITS2 | USA |
| FJ230355.1 | ITS2 | USA |
| FJ230356.1 | ITS2 | USA |
| FJ230357.1 | ITS2 | USA |
| FJ230358.1 | ITS2 | USA |
| FJ230359.1 | ITS2 | USA |
| FJ230360.1 | ITS2 | USA |
| FJ230361.1 | ITS2 | USA |
| FJ230362.1 | ITS2 | USA |
| FJ230363.1 | ITS2 | USA |
| FJ230364.1 | ITS2 | USA |
| FJ230365.1 | ITS2 | USA |
| FJ230366.1 | ITS2 | USA |
| FJ230367.1 | ITS2 | USA |
| FJ230368.1 | ITS2 | USA |
| FJ230369.1 | ITS2 | USA |
| FJ230370.1 | ITS2 | USA |
| FJ230371.1 | ITS2 | USA |
| FJ230372.1 | ITS2 | USA |
| FJ230373.1 | ITS2 | USA |
| FJ230374.1 | ITS2 | USA |
| FJ230375.1 | ITS2 | USA |
| FJ230376.1 | ITS2 | USA |
| FJ230377.1 | ITS2 | USA |
| FJ230378.1 | ITS2 | USA |
| FJ230379.1 | ITS2 | USA |
| FJ230380.1 | ITS2 | USA |
| FJ230381.1 | ITS2 | USA |
| GQ153611.1 | ITS2 | China |
| GQ153612.1 | ITS2 | China |
| GQ153613.1 | ITS2 | China |
| GQ153614.1 | ITS2 | China |
| GQ153615.1 | ITS2 | China |
| GQ153616.1 | ITS2 | China |
| GQ153617.1 | ITS2 | China |
| GQ153618.1 | ITS2 | China |
| GQ153619.1 | ITS2 | China |
| GQ153620.1 | ITS2 | China |
| GQ153621.1 | ITS2 | China |
| GQ153622.1 | ITS2 | China |
| GQ153627.1 | ITS2 | China |
| JF927793.1 | ITS2 | China |
| JN022586.1 | ITS2 | China |
| JN022587.1 | ITS2 | China |
| JN022588.1 | ITS2 | China |
| JN022589.1 | ITS2 | China |
| JN022590.1 | ITS2 | China |
| JN022591.1 | ITS2 | China |
| JN113571.1 | ITS2 | China |
| JN113572.1 | ITS2 | China |
| JN113573.1 | ITS2 | China |
| JN113574.1 | ITS2 | China |
| JN113575.1 | ITS2 | China |
| JN113576.1 | ITS2 | China |
| JN113577.1 | ITS2 | China |
| JN113578.1 | ITS2 | China |
| JN236214.1 | ITS2 | China |
| JN236215.1 | ITS2 | China |
| JF927793 | ITS1-5.8S rDNA-ITS2 | China |
| JN022586 | ITS1-5.8S rDNA-ITS2 | China |
| JN022587 | ITS1-5.8S rDNA-ITS2 | China |
| JN022588 | ITS1-5.8S rDNA-ITS2 | China |
| JN022589 | ITS1-5.8S rDNA-ITS2 | China |
| JN113571 | ITS1-5.8S rDNA-ITS2 | China |
| JN113572 | ITS1-5.8S rDNA-ITS2 | China |
| JN113573 | ITS1-5.8S rDNA-ITS2 | China |
| JN113575 | ITS1-5.8S rDNA-ITS2 | China |
| JN113576 | ITS1-5.8S rDNA-ITS2 | China |
| JN113577 | ITS1-5.8S rDNA-ITS2 | China |
| AY779388 | ITS1-5.8S rDNA-ITS2 | USA |
| AY779396 | ITS1-5.8S rDNA-ITS2 | USA |
| AY779400 | ITS1-5.8S rDNA-ITS2 | USA |
| AY779415 | ITS1-5.8S rDNA-ITS2 | USA |
| AY779420 | ITS1-5.8S rDNA-ITS2 | USA |
| AY779428 | ITS1-5.8S rDNA-ITS2 | USA |
| AY779439 | ITS1-5.8S rDNA-ITS2 | USA |
| AY779458 | ITS1-5.8S rDNA-ITS2 | USA |
| AY779468 | ITS1-5.8S rDNA-ITS2 | USA |
| AY779472 | ITS1-5.8S rDNA-ITS2 | USA |
| AY779475 | ITS1-5.8S rDNA-ITS2 | USA |
| AY779485 | ITS1-5.8S rDNA-ITS2 | USA |
| AY779489 | ITS1-5.8S rDNA-ITS2 | USA |
| AY779491 | ITS1-5.8S rDNA-ITS2 | USA |
| AY779504 | ITS1-5.8S rDNA-ITS2 | USA |
| AY779506 | ITS1-5.8S rDNA-ITS2 | USA |
| AY779509 | ITS1-5.8S rDNA-ITS2 | USA |
| AY779513 | ITS1-5.8S rDNA-ITS2 | USA |
| AY779514 | ITS1-5.8S rDNA-ITS2 | USA |
| FJ230318 | ITS1-5.8S rDNA-ITS2 | USA |
| FJ230319 | ITS1-5.8S rDNA-ITS2 | USA |
| FJ230328 | ITS1-5.8S rDNA-ITS2 | USA |
| FJ230337 | ITS1-5.8S rDNA-ITS2 | USA |
| FJ230339 | ITS1-5.8S rDNA-ITS2 | USA |
| FJ230341 | ITS1-5.8S rDNA-ITS2 | USA |
| FJ230358 | ITS1-5.8S rDNA-ITS2 | USA |
| FJ230360 | ITS1-5.8S rDNA-ITS2 | USA |
| FJ230366 | ITS1-5.8S rDNA-ITS2 | USA |
| FJ230372 | ITS1-5.8S rDNA-ITS2 | USA |
| FJ230373 | ITS1-5.8S rDNA-ITS2 | USA |
| FJ230380 | ITS1-5.8S rDNA-ITS2 | USA |
|  |  |  |
| Summary | ITS2: Australia | 32 |
|  | ITS2: China | 30 |
|  | ITS2: USA | 195 |
|  | ITS1-5.8S rDNA-ITS2 Australia | 0 |
|  | ITS1-5.8S rDNA-ITS2 China | 11 |
|  | ITS1-5.8S rDNA-ITS2 USA | 31 |
